# Determinants affecting utilisation of health services and treatment for children under-5 in rural Nepali health centres: a cross-sectional study

**DOI:** 10.1186/s12889-022-14318-y

**Published:** 2022-10-20

**Authors:** Binod Shrestha, Dan J. Green, Manish Baidya, Tim Chater, Jiban Karki, Andrew CK Lee, Seema Khadka, Gerda Pohl, Rudra Neupane, Simon Rushton

**Affiliations:** 1 PHASE Nepal, Suryabinayak 4, Bhaktapur, Nepal; 2 College of Health and Life Sciences, Aston University, B4 7ET Birmingham, UK; 3 Liverpool Clinical Trials Centre, University of Liverpool, L69 3BX Liverpool, UK; 4 International Public Health Department, Liverpool School of Tropical Medicine, L3 5QA Liverpool, UK; 5 School of Health and Related Research (ScHARR), University of Sheffield, S1 4DA Sheffield, UK; 6 Department of Politics and International Relations, University of Sheffield, S10 2TU Sheffield, UK

**Keywords:** Nepal, Social inequalities, Health inequalities, Child health, Treatment, Health-seeking behaviour, Global health

## Abstract

**Background:**

Large inequalities in child health remain in Nepal, with caste, ethnicity and sex being major determinants of deprivation and negative outcomes. The purpose of this study was to explore whether key demographics of under 5s were associated with health seeking behaviours, utilisation of health care, and treatment received.

**Methods:**

Data came from Integrated Management of Neonatal & Childhood Illness (IMNCI) records of 23 health centres across five districts. After digitising the paper records, the data was analysed by district, caste/ethnicity, sex, and age to investigate differences in the time taken to present at a health facility after the onset of symptoms of ARI, diarrhoea and fever; accuracy of diagnosis for pneumonia; and whether the correct treatment was prescribed for pneumonia as per IMNCI guidelines.

**Results:**

From 116 register books spanning 23 health centres, 30,730 child patient records were considered for analysis. The median age of attendance was 18 months (Inter-Quartile Range = 10, 32), while were more male children that attended (55.7% vs. 44.3% for females). There were statistically significant differences for the time taken to attend a health centre between different districts for ARI, diarrhoea and fever, with children in the remote Humla and Mugu districts taking significantly longer to present at a health facility after the onset of symptoms (all p < 0.001, except Mugu for ARI days). Children from underprivileged ethnic groups, Madhesi and Dalit, were less likely to be given a correct diagnosis of pneumonia (p = 0.014), while males were more likely to receive a correct diagnosis than females (73% vs. 67%, p = 0.001). This sex difference remained in the adjusted regression models for diagnosis of pneumonia (p < 0.001) but not for treatment of pneumonia (p = 0.628). All districts, in comparison to Gorkha, had increased odds of correct diagnosis and treatment of pneumonia, but only significant in children from Mugu after adjustment (p ≤ 0.001).

**Conclusion:**

Significant demographic differences were found based on ethnicity, sex, and district when examining health seeking behaviours for ARI, diarrhoea, and fever. Significant associations were seen for these same factors when exploring accuracy of diagnoses of pneumonia, but not for treatment. This study has emphasised the importance of a digitalised healthcare system, where inequalities can be identified without the reliance on anecdotal evidence.

**Supplementary Information:**

The online version contains supplementary material available at 10.1186/s12889-022-14318-y.

## Background

Despite considerable government efforts in recent years, Nepal continues to have huge social and geographic inequalities in child health. Nepal currently ranks 110th on the United Nations Development Programme’s Gender Inequality Index [1], and caste and ethnicity continue to be major determinants of poverty, education and health outcomes [2–5]. There are significant inequalities in access to and utilisation of basic health services between urban and rural areas, as well as between lowland, hill and mountain zones [2, 3, 5–7]. This presents a significant challenge for Nepal in terms of promoting social and economic development, meeting the Sustainable Development Goals to end preventable deaths of newborns and children under 5 years of age, and achieving universal health coverage.

Although work with the support of the World Health Organisation (WHO) on implementing electronic health management and information system (HMIS) is ongoing, one major shortcoming of current health data in Nepal is that individual patient-level data is not available at the government level: only aggregated data are reported from health facilities to the relevant provincial government. In large part, this is because health facilities, especially those in rural areas where infrastructure and capacity are lacking, continue to rely on paper-based health records which are never digitised. Consequently, individual patient records cannot easily be investigated for health outcome correlations with gender, ethnicity and caste [7]. For the same reason, there is a significant data gap that prevents monitoring and evaluation of the practices of frontline health workers, such as the extent to which they comply with treatment guidelines [7, 8].

Previous research investigating inequalities in health-seeking behaviour, diagnosis and treatment in Nepal has, as a result, been based upon either health-seeking behaviours self-reported via household surveys [9, 10] or data from Nepal’s Demographic Health Survey [11–17], and has not (to our knowledge) previously taken advantage of facility-level data. It has tended to focus on adult patients, in particular on maternal health services [9, 14, 16, 18] and the management of hypertension [13, 15]. That literature provides ample reason to believe that direct or indirect discrimination based on gender, caste, geography and other factors including level of education and household income have significant impacts on healthcare utilisation, and also on the quality of care received when visiting a health facility [11, 12, 15, 17, 19]. Research has shown, for example, that health services commonly fail to reach low castes, ethnic minorities, people living in remote areas, and other marginalized populations [20].

Health care utilization by under-5s, the focus of this study, depends on the health-seeking behaviors of parents and caregivers. As seen in studies elsewhere in South Asia, parents and caregivers do not always adequately seek health care services for childhood illness for a variety of reasons, including a preference for traditional healers [21, 22], low levels of education[23], and living long distances from a health center [21, 23]. Health-seeking patterns for children have also been found to be highly gendered in developing countries [24, 25]. Resultant delays in care-seeking can make illness more severe, or in some cases mean that the child does not receive treatment at all. Significant disparities have been found according to class, caste, ethnicity, and literacy in an assessment of health utilization and health service provision done by a health improvement program in Nepal [26].

Enhanced evidence on how inequalities affect health status, health service utilisation, and treatment provided, and the extent to which current health worker practices either mitigate or reinforce those inequalities, is highly pertinent for academic, policy and practice audiences. A good understanding of the impacts of inequality on health-seeking behaviours and healthcare will allow for the co-production of better policy and practice recommendations. This study sought to explore how age, sex, ethnicity and caste of young children affect: (1) their health-seeking behaviours and utilisation of health services; and (2) the treatment that they receive at health centres, with a particular focus on diagnosis and prescribing practices. A secondary objective of the study was to examine the utility of health data that is routinely collected by rural health centres.

## Methods

The study was carried out by a team of researchers from the University of Sheffield in partnership with a Nepali non-governmental organisation (NGO), PHASE Nepal, which works in remote Himalayan communities to support health, education and livelihoods. We conducted a retrospective analysis of cross-sectional health data that is routinely recorded by rural health centres.

### Setting

The data for this project comes from information recorded and stored as paper records at 23 health centres supported (or previously supported) by PHASE Nepal. The funding for the project (from the GCRF NGO Secondary Data Initiative) was specifically intended to support the systematisation and analysis of data held by NGOs that had not previously been available for analysis by researchers. As such, the project’s data sampling included data from the 23 government health posts that were being supported (or had recently been supported) by PHASE Nepal.

PHASE Nepal specialises in working in some of the most remote regions of the country, where government services are weak and other NGO support is limited or entirely absent. The 23 health centres involved in the study are spread across 5 Districts, all of which are in the Hill or Mountain zones of Nepal; Bajura, Humla and Mugu in the Far West of the country; and the central districts of Gorkha and Sindhupalchok. The three districts in the Far West are very remote with limited road access, services, and amenities. Wealth quintiles and literacy rates vary significantly between the two regions. About half of the population in the Far West lives below the poverty line. The Far West region has complex socio-economic structures and there is widespread gender- and caste-based discrimination. The central region is more varied, being home to various ethnic communities including Newar, Tamang, and Brahmins as well as different religions, including Hinduism, Buddhism and Islam. Levels of gender- and caste-based discrimination vary widely between different communities. All district headquarters in the central region have road connectivity, but geographical isolation, vulnerable roads and a lack of bridges across many rivers are nevertheless major challenges in the central region.

### Data collection

Research Assistants visited each of these centres to collect the data. The paper records were either digitally photographed and transported on secure electronic media to the PHASE Nepal office in the Kathmandu valley, or the record books themselves were taken to the PHASE Nepal office, photocopied, and returned to the health centres. All records were stored securely at the PHASE Nepal offices, as per the project’s Data Management Plan.

### Description of data

The record books utilised in this study record individual patient contacts for children under 5 years of age. The format of the record books follows the WHO (Nepal) guidance on Community-Based Integrated Management of Neonatal and Childhood Illness (IMNCI).

The data captured on the forms include the following information: a unique identifier, date of visit, child’s sex, age, ethnicity/caste, weight, temperature, symptoms of general danger signs (GDS), symptoms of acute respiratory infection (ARI) (including respiratory rate), diarrhoea and dehydration symptoms, symptoms for fever and ear infections, mid upper arm circumference (MUAC) measurement, assessment of nutritional status, classification for one major diagnosis, medicine prescribed (name of medicine), follow up plan, and condition of child on the date of follow up.

There were two main versions of the register books in use across the 23 health centres: Integrated Management of Childhood Illness (IMCI) and IMNCI. The two versions of the register books were broadly similar, although there was some variation in the precise format of the forms (e.g. slight differences in the questions or slight differences in the answer options provided in different registers). There were separate forms for children from age two to 59 months, and children below two months of age. The differences in questions and answer choices in each type of register are displayed in Supplementary Fig. 1. The data digitisation tool was designed to ensure that the data entered from the slightly different books adhered to a common format, with no loss or amendment of data. In addition, detailed guidelines were devised by the research team to guide data input and analysis, to ensure that where the form formats differed, they were consistently and accurately mapped onto the data digitisation tool.

### Digitisation of data

A team of 12 trained Data Entry Assistants entered the data from the images of the paper records into a bespoke data digitisation tool (using KoBo Toolbox [27]), which was designed by members of the project team. At this stage, all data were pseudonymised and referred to only by a code number: direct identifiers (such as names and addresses, as well as any other information that could lead to the identification of an individual patient) were not entered into the database.

### Data quality checks

A number of measures were taken to ensure a high standard of data quality. *Point-of-entry validation* was enforced by checks carried out by the digitisation tool during data entry to ensure mandatory fields were completed, and to prevent entry of invalid data. *Data entry verification* was conducted by reviewing a sample (5%) of entered records against the original digital image and any data entry errors were noted and corrected. If consistent errors were identified, this was investigated and all potentially affected records were checked and, where necessary, corrected. *Post-entry validation* consisted of checks of the data which had already been entered against a set of pre-defined rules to identify potentially invalid, out-of-range, inconsistent or missing data. Where data was not compliant with the validation rules, the discrepancies were highlighted and subsequently reviewed and resolved.

### Participants and excluded data

The IMNCI register books were identified from the sampled health facilities, dated from 2068/05/07 BS (24 August 2011) to 2077/08/12 BS (27 November 2020). Records were collected from 23 rural health posts included in the study. Registers should only be completed for children up to five years of age (< 60 months); in the event a child’s age was declared as equal to or greater than 60 months, or the age value was missing, the record was excluded from the analysis. For the main analysis we also excluded children whose ages were declared as under 2 months, as we wanted to focus the research on those aged 2 to 59 months. Where the child’s sex was not recorded in the register, the sex was inferred based on the child’s first name where possible, otherwise it was left as missing. As a sensitivity analysis, we examined the impact of leaving all the undeclared sexes as missing.

### Health seeking behaviour and service utilisation

To assess health seeking behaviour and utilisation of health services, we analysed the number of days the child had symptoms before visiting the health facility for one of the four conditions of interest: acute respiratory infection, diarrhoea, fever, and ear infection. We initially intended to also investigate whether the child attended the health centre for a follow-up; however, as the Master Registration Number field was not used consistently, it became impossible to track follow-up cases with a lack of data linkage for patients between different clinic attendees.

### Assessment of diagnostic accuracy and appropriateness of care

We assessed the diagnostic accuracy for pneumonia recorded by health workers by comparing their diagnoses with our retrospective diagnosis using the IMNCI case definition for pneumonia based on the presenting symptoms. We then assessed the appropriateness of treatment for pneumonia by comparing the recorded treatment provided by health workers to children whom they diagnosed as having pneumonia with the IMNCI treatment guidelines.

## Statistical methods

Descriptive statistics were summarised using count (percentage) and median with Inter-Quartile Range (IQR). The most appropriate hypothesis test was performed to explore unadjusted associations depending on the variable types and distributions (indicated in the footnote of Table 1). To account for the impact of confounding variables, multivariable linear regression, with robust standard errors to produce more conservative estimates, was used to assess the number of symptom days a child had ARI, diarrhoea or fever before consultation (ear infection was not modelled due to small numbers). The main predictors of age, sex, ethnicity and district were included in the multivariable model regardless of their univariable significance; the remaining predictors (temperature, whether child was referred to clinic, GDS, ARI, diarrhoea, ear infection and fever) were only included in the multivariable model if their univariable significance was p < 0.20. Multivariable logistic regression was used to investigate the relationship between demographics and whether the appropriate pneumonia diagnosis and subsequent antibiotic prescription had been provided or not.

**Table 1:** *Median time from symptom onset to attendance at a health centre for young children (age 2–59 months) with ARI, Diarrhoea, Fever and Ear Infection*

	ARI			Diarrhoea			Fever			Ear Infection		
	**n**	**Med (IQR)**	**p**	**n**	**Med (IQR)**	**p**	**n**	**Med (IQR)**	**p**	**n**	**Med (IQR)**	**p**
**District**			< 0.001			< 0.001			< 0.001			0.350
Sindhupalchowk	1377	3 (2, 3)		638	2 (2, 3)		1449	2 (2, 3)		1	2 (2, 2)	
Gorkha	1477	2 (2, 3)		1351	2 (2, 3)		1619	2 (2, 3)		0	-	
Humla	769	3 (2, 5)		994	3 (2, 5)		721	3 (2, 4)		0	-	
Mugu	1144	3 (3, 5)		1976	3 (3, 5)		1238	3 (2, 4)		0	-	
Bajura	3679	3 (2, 3)		3480	3 (2, 3)		3481	3 (2, 3)		49	3 (2, 4)	
**Ethnicity**												
Dalit	1838	3 (2, 4)	< 0.001	2007	3 (2, 4)	< 0.001	1913	3 (2, 3)	< 0.001	11	3 (2, 5)	0.829
Janajati	2655	2 (2, 3)		2002	2 (2, 3)		2877	2 (2, 3)		2	5 (2, 7)	
Madhesi	41	3 (2, 4)		54	3 (3, 3)		43	3 (2, 3)		0	-	
Muslim	13	3 (2, 3)		11	3 (2, 6)		12	3 (2.5, 3)		0	-	
Brahmin/ Chhetri	2885	3 (2, 4)		3212	3 (2, 4)		2828	3 (2, 3)		19	3 (2, 3)	
Others	931	3 (2, 4)		1103	3 (2, 5)		795	3 (2, 3)		18	3 (2, 5)	
**Sex**			0.820			0.945			0.111			0.590
Female	3717	3 (2, 3)		3709	3 (2, 4)		3737	2 (2, 3)		21	3 (2, 3)	
Male	4714	3 (2, 3)		4721	3 (2, 4)		4760	2 (2, 3)		29	3 (2, 4)	
**Total**	8446	3 (2, 3)		8439	3 (2, 4)		8508	2 (2, 3)		50	3 (2, 4)	

*ARI = Acute Respiratory Infection; IQR = Inter-Quartile Range; All p-values either Mann-Whitney U tests, or Kruskal Wallis ANOVA depending whether comparing 2 or 3 groups*

All regression models were analysed as random-effects taking account for the potential clustering of the health facility. In addition to this, multiple imputation (MI) using chained equations was used to impute missing values in all the predictors of interest, with blocks of ten imputations in each regression model, as the primary analysis. Multicollinearity was assessed using the variance inflation factor, with scores higher than 10 investigated further. Specific pair-wise interactions with gender and other factors were investigated in the regression models. As a sensitivity analysis, we also repeated all the regression analyses using only complete case analysis; all sensitivity analyses are presented as supplementary tables.

All inferential analysis is presented with two-sided p-values, where p < 0.05 was considered statistically significant, and with 95% confidence intervals. Analyses were performed in Stata version 15.0 [28]. The statistical analysis plan was not pre-registered, therefore all analyses should be considered explanatory.

## Results

**Fig. 1:**
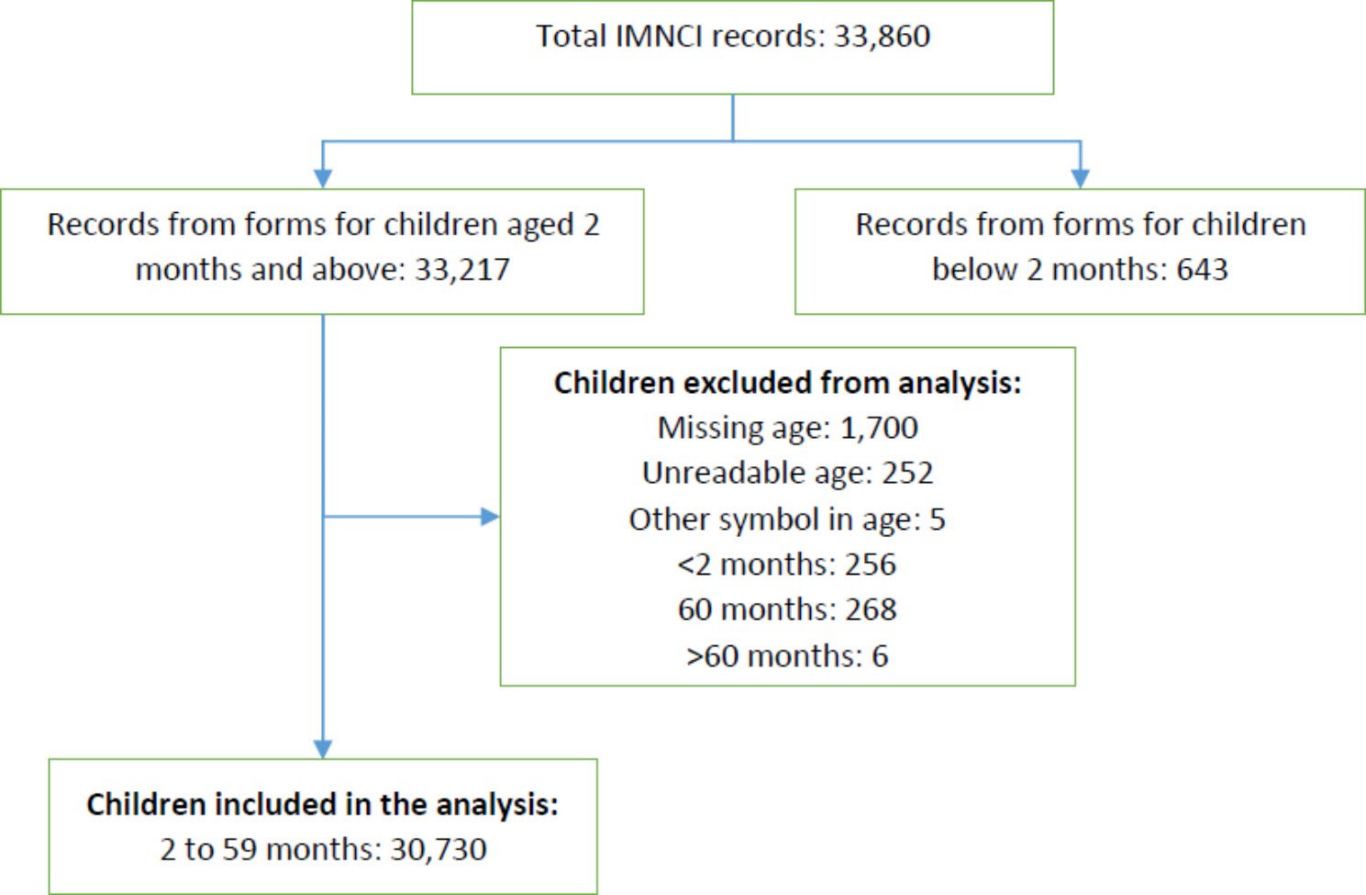
Flow diagram illustrating the number of records identified, and those carried forward for analysis, with reason for exclusion noted

There were a total of 33,860 child patient records retrieved from a total of 116 IMNCI register books from the 23 rural health posts. Of this total, 643 (1.9%) child patient records from IMNCI forms for children below the age of 2 months were excluded from the analysis. 33,217 records were for children aged 2 to 59 months. 274 (0.8%) records had a recorded age equal to or greater than 60 months, 1,957 (5.9%) records had missing or unclear age recorded and 256 (0.8%) records had patient age stated as below 2 months recorded in the 2 to 59 months register; these were all excluded from the analysis. Therefore, 30,730 records were taken forward for analysis (Fig. 1).

The median age of children was 18 months (IQR = 10, 32 months) (Table 2). There were more attendances recorded for male children than female children (55.7% vs. 44.3%). In terms of ethnic groups, the highest proportion of attendances was for children with Janajati ethnicity (34.9%), followed by Brahmin/Chhetri (32.7%). Madhesi and Muslim children comprised only 0.7% combined, which would be expected given the location of the health centres and the geographical distribution of Madhesi and Muslim population groups, who are mainly concentrated in the Terai (lowland areas) that were not part of this study.

**Table 2:** Overall demographics for all records collected, all frequency and percentage unless otherwise stated

n = 30,730→			
Age (months) (2–59)*	**Median, IQR (n = 30,730)**	**18 months**	**10, 32 months**
Sex	Female	13,617	44.3%
	Male	17,113	55.7%
Weight, kg	n = 26,268; Mean, SD	9.54	2.87
Temperature (^o^C)	n = 15,224; Mean, SD	37.04	0.89
District	Sindhupalchowk	3,929	12.8%
	Gorkha	7,271	23.7%
	Humla	3,371	11.0%
	Mugu	4,215	13.7%
	Bajura	11,944	38.9%
Visit year	2068 to 2073 (2011/12 to 2016/17)	10,169	33.1%
	2074 to 2077 (2017/18 to 2020/21)	20,561	64.1%
	Unreadable	133	0.4%
	Missing	761	2.5%
Ethnicity	Dalit	6,409	20.9%
	Janajati	10,716	34.9%
	Madhesi	159	0.5%
	Muslim	70	0.2%
	Brahmin/Chhetri	10,050	32.7%
	Others (Thakuri, Sanyasi/Dasnami)	2,987	9.7%
	Missing	321	1.0%
	Unreadable	27	0.1%
Referred by	CHW	79	0.3%
	HF	863	2.8%
	None	10,348	33.7%
	FCHV	15	0.1%
	PHC/ORC	39	0.1%
	Missing	19,386	63.1%

*= all children with a valid age recorded; ARI = Acute Respiratory Infection; CHW = Community Health Worker; FCHV = Female Community Health Worker; GDS = General Danger Sign; HF = Health Facility; IQR = Inter-Quartile Range; kg = kilograms; °C *= degrees Celsius; ORC = Outreach Clinic; PHC = Primary Health Centre; SD = Standard Deviation*

42% of children were recorded as having fever, 37% with diarrhoea, and 34% with respiratory symptoms. Less than 7% of children were recorded as having an ear infection, while 2.1% had General Danger Signs (GDS).

### Days of illness prior to presentation at a health centre

The time interval between onset of symptoms and attendance at a health centre for children with ARI was lowest in Gorkha District with a median of 2 days (IQR = 2, 3) (Table 1). There were significant differences in time to attendance between the five districts for ARI, diarrhoea and fever with longer delays for Bajura, Mugu and Humla (in the Far West) compared to the other central districts. Janajati children were more likely to attend the health post sooner than children from other ethnic groups for all conditions with the exception of ear infections (p < 0.001). There were no significant differences between a child’s sex and duration of symptoms prior to attendance for each condition.

The records from the years 2068 BS (2011/12 AD) (n = 43), 2069 BS (2012/13 AD) (n = 9), and 2077 BS (2020/21 AD) (n = 77) were removed from the regression modelling due to convergence issues. The unadjusted and adjusted regression results for the number of days a child had ARI, diarrhoea or fever before visiting a health post are presented in Tables 3, 4 and 5 respectively. For ARI, only the Janajati ethnic group was significant in the unadjusted model (p < 0.001) compared to Dalit children, while male Janajati and Brahmin/Chherti children with ARI waited on average more than half a day less than females (interaction coef=-0.55, p = 0.025 and coef=-0.73, p = 0.001 respectively) (Table 3). Children with ARI from Humla in the Far West visited the health post on average one day later than those from Gorkha (1.05, 95%CI: 0.44, 1.65).

**Table 3:** *Univariable and multivariable linear regression of number of ARIs day using multiple imputation analysis*

	ARI (n = 8651)					
	**Coef**	**95% CI**	**P-Value**	**Adj Coef**	**95% CI**	**P-Value**
**Health facility district**						
Gorkha	REF			REF		
Sindhupalchowk	0.17	-0.32, 0.66	0.494	0.72	0.18, 1.26	0.009
Humla	0.94	0.41, 1.47	0.001	1.05	0.44, 1.65	0.001
Mugu	1.18	0.85, 1.52	< 0.001	0.38	-0.26, 1.03	0.240
Bajura	0.65	0.09, 1.20	0.022	0.72	0.18, 1.26	0.009
**Visit year Nepali year**						
2070 to 2073	REF			REF		
2074 to 2076	-0.40	-0.81, 0.01	0.058	-0.30	-0.69, 0.09	0.134
**Ethnicity**						
Dalit	REF			REF		
Janajati	-0.68	-1.03, -0.32	< 0.001	-0.18	-0.85, 0.49	0.602
Brahmin/Chhetri	-0.09	-0.40, 0.22	0.567	0.21	-0.25, 0.66	0.375
Others	-0.26	-0.48, -0.04	0.021	-0.47	-0.76, -0.17	0.002
**Sex**						
Female	REF			REF		
Male	-0.15	-0.33, 0.02	0.086	0.27	-0.11, 0.64	0.162
**Age (months)**	-0.01	-0.02, -0.00	0.002	-0.01	-0.01, 0.00	0.087
**Temperature (°C)**	-0.30	-0.49, -0.10	0.003	-0.26	-0.47, -0.05	0.015
**Referred**						
No	REF			REF		
Yes	-0.41	-0.84, 0.01	0.055	-0.35	-0.79, 0.09	0.119
**GDS**						
No	REF			REF		
Yes	1.03	-0.19, 2.24	0.097	0.98	-0.20, 2.16	0.102
**Diarrhoea**						
No	REF			REF		
Yes	0.42	0.15, 0.68	0.002	0.29	0.02, 0.56	0.034
**Ear Infection**						
No	REF					
Yes	0.15	-0.61, 0.92	0.681			
**Fever**						
No	REF			REF		
Yes	-0.37	-0.67, -0.06	0.018	-0.14	-0.47, 0.20	0.428
Interaction: Gender*Ethnicity						
Male Dalit				-0.10	-0.54, 0.34	0.659
Male Janajati				-0.55	-1.03, -0.07	0.025
Male Brahmin/Chhetri				-0.73	-1.17, -0.29	0.001

*Also adjusted for visit year; Muslim and Madhesi removed due to sample size; ARI = Acute Respiratory Infection; CI = Confidence Interval;* °C *= degrees Celsius; GDS = General Danger Sign; REF = Reference category*

**Table 4:** *Univariable and multivariable linear regression of number of diarrhoea days using multiple imputation analysis*

	Diarrhoea (n = 8660)					
	**Coef**	**95% CI**	**P-Value**	**Adj Coef**	**95% CI**	**P-Value**
**Health facility district**						
Gorkha	REF			REF		
Sindhupalchowk	0.12	-0.29, 0.53	0.567	0.36	-0.18, 0.90	0.195
Humla	2.00	1.38, 2.62	< 0.001	1.86	1.47, 2.25	< 0.001
Mugu	2.00	1.57, 2.43	< 0.001	1.79	1.12, 2.46	< 0.001
Bajura	0.86	0.25, 1.47	0.006	0.79	-0.04, 1.62	0.061
**Visit year Nepali year**						
2070 to 2073	REF			REF		
2074 to 2076	0.07	-0.26, 0.41	0.668	0.01	-0.31, 0.32	0.971
**Ethnicity**						
Dalit	REF			REF		
Janajati	-1.20	-1.69, -0.70	< 0.001	-0.42	-0.68, -0.15	0.002
Brahmin/Chhetri	-0.40	-0.85, 0.04	0.072	-0.46	-0.89, -0.02	0.039
Others	-0.66	-1.18, -0.15	0.011	-0.77	-1.31, -0.23	0.005
**Sex**						
Female	REF			REF		
Male	0.02	-0.12, 0.15	0.824	0.04	-0.14, 0.23	0.655
**Age (months)**	-0.01	-0.02, 0.00	0.110	-0.01	-0.02, 0.00	0.271
**Temperature (°C)**	-0.23	-0.43, -0.03	0.027	-0.23	-0.43, -0.03	0.022
**Referred**						
No	REF					
Yes	0.39	-0.36, 1.13	0.305			
**GDS**						
No	REF					
Yes	0.44	-0.59, 1.47	0.406			
**ARI**						
No	REF					
Yes	-0.03	-0.23, 0.17	0.758			
**Ear Infection**						
No	REF					
Yes	-0.02	-0.81, 0.76	0.949			
**Fever**						
No	REF					
Yes	-0.07	-0.50, 0.36	0.758			
Interaction: Gender*District						
Male Sindhupalchowk				-0.52	-0.96, -0.08	0.020
Male Gorkha				0.00	-0.29, 0.28	0.987
Male Humla				0.03	-0.61, 0.67	0.928
Male Mugu				0.05	-0.21, 0.31	0.695

*Also adjusted for visit year; Muslim and Madhesi removed due to sample size; ARI = Acute Respiratory Infection; CI = Confidence Interval;* °C *= degrees Celsius; GDS = General Danger Sign; REF = Reference category*

**Table 5:** *Univariable and multivariable linear regression of number of fever days using multiple imputation analysis*

	Fever (n = 2462)					
	**Coef**	**95% CI**	**P-Value**	**Adj Coef**	**95% CI**	**P-Value**
**District**						
Gorkha	REF			REF		
Sindhupalchowk	-0.01	-0.23, 0.21	0.936	0.05	-0.19,0.30	0.661
Humla	1.24	0.60, 1.89	< 0.001	1.53	0.83,2.24	< 0.001
Mugu	0.84	0.48, 1.20	< 0.001	0.88	0.57,1.19	< 0.001
Bajura	0.59	0.27, 0.92	< 0.001	0.59	0.25,0.93	0.001
**Visit year Nepali year**						
2070 to 2073	REF			REF		
2074 to 2076	-0.10	-0.41,0.20	0.506	-0.11	-0.41,0.18	0.455
**Ethnicity**						
Dalit	REF			REF		
Janajati	-0.41	-0.65,-0.17	0.001	-0.13	-0.33,0.08	0.224
Brahmin/Chhetri	-0.01	-0.20,0.17	0.879	-0.03	-0.20,0.15	0.776
Others	-0.06	-0.46,0.34	0.774	-0.10	-0.52,0.32	0.632
**Sex**						
Female	REF			REF		
Male	-0.14	-0.27,-0.01	0.034	0.02	-0.11,0.14	0.797
**Age (months)**	0.00	-0.00,0.00	0.596	0.00	-0.00,0.01	0.156
**Temperature (°C)**	-0.07	-0.17,0.03	0.143	-0.08	-0.17,0.02	0.131
**Referred**						
No	REF					
Yes	-0.19	-0.49,0.12	0.227			
**GDS**						
No	REF			REF		
Yes	1.16	-0.18,2.50	0.089	1.11	-0.24,2.45	0.106
**ARI**						
No	REF			REF		
Yes	0.18	0.03,0.33	0.017	0.17	0.03,0.31	0.017
**Diarrhoea**						
No	REF			REF		
Yes	0.18	0.01,0.36	0.040	0.12	-0.08,0.32	0.254
**Ear Infection**						
No	REF					
Yes	0.09	-0.23,0.40	0.596			
Interaction: Gender*District						
Male Sindhupalchowk				-0.04	-0.19,0.11	0.602
Male Gorkha				-0.66	-1.60,0.27	0.162
Male Humla				-0.20	-0.57,0.16	0.273
Male Mugu				-0.15	-0.32,0.02	0.084

*Also adjusted for visit year; Muslim and Madhesi removed due to sample size; ARI = Acute Respiratory Infection; CI = Confidence Interval;* °C *= degrees Celsius; GDS = General Danger Sign; REF = Reference category. NB. The regression residuals were slightly bi-modal for the multivariable fever model, and while transformations were attempted to improve the error, they only made the fit worse*

The difference in time taken to seek help for diarrhoea in those from the Far West (Humla and Mugu) was significant in both the unadjusted and adjusted models (both p < 0.001), and a strong, albeit non-significant, association for Bajura district (also in the Far West) (p = 0.061), Table 4. Children with diarrhoea in Mugu and Humla waited on average 2 days longer to visit the health post (1.86, 95% CI: 1.47, 2.25 and 1.79, 95%CI: 1.12, 2.46 respectively) than those in Gorkha.

District, and ARI were significantly associated with time delay to presentation for fever in the adjusted model (both p ≤ 0.02, Table 5) while there was a non-significant, but associative interaction for male children from Mugu to attend sooner than females (p = 0.08). Children with fever from Humla (1.53, 95% CI: 0.83, 2.24), Mugu (0.88, 95% CI: 0.57, 1.19) and Bajura (0.59, 95% CI: 0.25, 0.93) waited significantly longer than those from Gorkha.

As mentioned previously, due to issues with accuracy of data completion for follow-up, it was not possible to formally analyse this part of the information. The main reasons were that children were infrequently allocated a Master Registration Number (MRN), akin to a unique ID for that child, and even when they were, there were issues of accuracy (sometimes the same number appeared for two different children of different sexes). We have included the information we could from the registers in Supplementary Table 1.

### Correct diagnosis and treatment of pneumonia

For the children for whom we were able to retrospectively diagnose pneumonia from their presenting symptoms, 22.0% had no pneumonia; 62.9% had pneumonia and 15.0% had severe pneumonia (based on IMNCI guidelines). However, among all children whose pneumonia was diagnosed by the health worker, 65.3% had no pneumonia, 34.0% had pneumonia and 0.7% had severe pneumonia. The accuracy of pneumonia diagnoses by the health worker could only be analysed for 2,548 children due to missing data in variables necessary to assess the diagnosis of pneumonia retrospectively from the records. Pneumonia was not correctly diagnosed in 30% of children. Children from the Madhesi and Dalit ethnic group were less likely to have a correct diagnosis of pneumonia (36.4% and 66.3% respectively). Male children were significantly more likely to be correctly diagnosed for pneumonia (73.3% vs. 67%).

Of the 2,663 children who were recorded as having severe pneumonia or pneumonia by the health worker, over 60% were not provided correct treatment in line with the IMNCI guidelines (Table 6).

**Table 6:** *Pneumonia correctly diagnosed and correct treatment provided by health worker*

	Pneumonia correctly diagnosed					Pneumonia correctly treated				
	No (n)	%	Yes (n)	%	P-value	No (n)	%	Yes (n)	%	P-value
**Ethnicity**										
Dalit	189	33.8	371	66.3	0.014*	305	59.0	212	41.0	0.185*
Janajati	197	29.1	479	70.9		507	65.9	262	34.1	
Madhesi	7	63.6	4	36.4		4	57.1	3	42.9	
Muslim	0	0.0	5	100.0		2	50.0	2	50.0	
Brahmin/Chhetri	283	28.1	724	71.9		663	64.0	373	36.0	
Others	72	27.1	194	72.9		188	63.3	109	36.7	
**Sex**										
Female	375	33.0	762	67.0	0.001	709	62.8	420	37.2	0.408
Male	375	26.7	1030	73.3		986	64.4	546	35.6	
**Referred**										
No	339	30.6	770	69.4	0.002	714	66.1	366	33.9	< 0.001
Yes	50	44.6	62	55.4		32	42.7	43	57.3	
**GDS**										
Yes	122	91.0	12	9.0	< 0.001	47	64.4	26	35.6	0.532
No	548	26.1	1554	74.9		1328	60.8	858	39.3	
**ARI**										
Yes	702	29.9	1645	70.1	0.599	1456	61.4	914	38.6	< 0.001
No	45	28.0	116	72.0		124	76.5	38	23.5	
**Diarrhoea**										
Yes	181	28.1	464	72.9	0.313	406	62.5	244	37.5	0.432
No	513	30.2	1186	69.8		1044	60.7	676	39.3	
**Ear Infection**										
Yes	33	41.3	47	58.8	0.013	45	65.2	24	34.8	0.305
No	588	28.4	1484	71.6		1246	59.1	864	41.0	
**Fever**										
Yes	444	26.7	1222	73.4	< 0.001	1055	59.6	715	40.4	< 0.001
No	254	34.9	474	65.1		449	67.8	213	32.2	
**Total**	**756**	**29.7**	**1792**	**70.3**		**1696**	**63.7**	**967**	**36.3**	

All p-values Chi-square test unless marked with a * (*=Fishers Exact Test); ARI = Acute Respiratory Infection; °C = degrees Celsius; GDS = General Danger Sign

Children in the Janajati group had the lowest proportion of correct treatment whereas the highest proportion receiving correct treatment was seen for Dalit children (34.1% vs. 41%). This excludes Madhesi and Muslim children that were not included in the subsequent regression analysis due to the small numbers.

Janajati children had twice the odds of a correct pneumonia diagnosis compared to Dalit children (Odds Ratio (OR) = 2.06, 95%CI: 1.16, 3.67) after adjusting for background factors, while children with higher body temperatures also had 19% higher odds (OR = 1.19, 95%CI: 1.01, 1.41) (Table 7). Males had significantly higher odds of a correct diagnosis than females (OR = 1.59, 95%CI: 1.27, 1.2.01). Males from “other” ethnicities had 49% lower odds of a correct diagnosis compared to females (OR = 0.51, 95%CI: 0.30, 0.85).

**Table 7:** *Unadjusted and adjusted logistic regression results of correct diagnosis of pneumonia vs. not (reference) and correct treatment of pneumonia vs. not (reference) using multiple imputation analysis*

	Correct diagnosis of Pneumonia (n = 2,611)						Correct treatment of Pneumonia (n = 1,739)					
	**OR**	**95% CI**	**P-Value**	**AOR**	**95% CI**	**P-Value**	**OR**	**95% CI**	**P-Value**	**AOR**	**95% CI**	**P-Value**
**District**												
Gorkha	REF			REF			REF			REF		
Sindhupalchowk	1.22	0.59, 2.53	0.584	1.19	0.57, 2.49	0.635	4.05	0.96, 17.19	0.058	5.48	0.96, 31.28	0.056
Humla	0.98	0.37, 2.64	0.974	1.43	0.44, 4.61	0.551	2.00	0.57, 6.95	0.276	3.92	0.87, 17.63	0.075
Mugu	3.34	1.48, 7.52	0.004	5.71	2.03, 16.10	0.001	7.68	2.38, 24.76	0.001	12.00	3.10, 46.50	< 0.001
Bajura	1.31	0.59, 2.91	0.500	1.73	0.60, 5.02	0.311	2.96	1.09, 8.00	0.032	3.44	0.98, 12.08	0.053
**Visit year Nepali year**												
2070 to 2073	REF			REF			REF			REF		
2074 to 2076	0.80	0.55, 1.16	0.233	0.73	0.48, 1.10	0.131	2.49	0.90, 6.87	0.077	1.81	0.69, 4.75	0.231
**Ethnicity**												
Dalit	REF			REF						REF		
Janajati	1.29	0.80, 2.07	0.293	2.06	1.16, 3.67	0.014	1.15	0.60, 2.18	0.679	1.22	0.65, 2.31	0.530
Brahmin/Chhetri	1.16	0.92, 1.46	0.197	1.29	0.86, 1.95	0.222	1.13	0.86, 1.49	0.391	1.16	0.86, 1.57	0.339
Others	0.90	0.71, 1.15	0.388	1.30	0.81, 2.07	0.274	0.87	0.47, 1.61	0.656	0.98	0.57, 1.67	0.939
**Sex**												
Female	REF			REF			REF			REF		
Male	1.36	1.12, 1.66	0.002	1.59	1.27, 2.01	< 0.001	0.89	0.66, 1.20	0.437	1.29	0.46, 3.63	0.628
**Age (months)**	1.00	0.99, 1.00	0.488	1.00	0.99, 1.01	0.469	1.00	0.99, 1.02	0.879	1.00	0.99, 1.01	0.952
**Temperature (°C)**	1.23	1.08, 1.41	0.002	1.19	1.01, 1.41	0.039	1.04	0.93, 1.16	0.520			
**Referred**												
No	REF						REF			REF		
Yes	0.97	0.66, 1.44	0.887				4.82	2.43, 9.56	< 0.001	3.98	2.23, 7.10	< 0.001
**GDS**												
No	REF			REF			REF					
Yes	0.03	0.01, 0.05	< 0.001	0.02	0.01, 0.05	< 0.001	1.28	0.17, 9.81	0.808			
**ARI**												
No	REF						REF					
Yes	0.84	0.54, 1.30	0.434				1.18	0.33, 4.23	0.796			
**Diarrhoea**												
No	REF						REF					
Yes	0.98	0.81, 1.19	0.824				0.93	0.69, 1.27	0.663			
**Ear Infection**												
No	REF			REF			REF					
Yes	0.51	0.31, 0.86	0.012	0.60	0.36, 1.00	0.049	0.58	0.26, 1.29	0.173	0.54	0.22, 1.31	0.167
**Fever**												
No	REF			REF			REF			REF		
Yes	1.57	1.22, 2.02	< 0.001	1.57	1.18, 2.09	0.002	1.32	0.89, 1.95	0.161	1.40	0.86, 2.27	0.176
Interaction: Gender*Ethnicity												
Male*Janajati				0.76	0.38, 1.53	0.442						
Male*Brahmin/ Chhetri				0.84	0.56, 1.26	0.406						
Male*Others				0.51	0.30, 0.85	0.011						
Interaction: Gender*District												
Male*Sindhupalchowk										0.59	0.20, 1.79	0.355
Male*Humla										0.37	0.13, 1.06	0.064
Male*Mugu										0.37	0.12, 1.09	0.070
Male*Bajura										0.86	0.29, 2.57	0.788

*Also adjusted for visit year; Muslim and Madhesi removed due to sample size; AOR = Adjusted Odds Ratio; ARI = Acute Respiratory Infection; CI = Confidence Interval;* °C *= degrees Celsius; GDS = General Danger Sign; OR = Odds Ratio; REF = Reference category*

Associations with correct treatment of pneumonia (given they had been diagnosed) were not so plentiful, with no significant difference in ethnicity, sex or age (all p > 0.3). The main significant relationship was if the child had been referred, with 4 times the odds (OR = 3.98, 95%CI: 2.23, 7.10) and a tendency for male children in the Mugu district having lower odds of a correct treatment compared to females, although not statistically significant (OR = 0.37, 95%CI: 0.12, 1.09).

Multicollinearity was not evident in any of the regression models, with all VIF < 10.

### Sensitivity analyses

The complete case regression models (Supplementary Tables 2–5) produced similar conclusions to the multiple imputation models, although the MI regression models frequently included more variables in the final multivariable model. Excluding all the 828 children who did have a sex declared as missing from the analysis only subtly changed the estimates and did not impact any conclusions.

## Discussion

In this large secondary analysis of healthcare records of children under 5 in Nepal, we have highlighted that there are some significant differences between health seeking behaviours and particular demographics such as District, ethnicity, and in some cases age. In addition, there appeared to be some more clear differences in achieving a successful diagnosis of pneumonia based on the child’s ethnicity, sex and District even after accounting for background factors; however, these differences were not as apparent when examining subsequent treatment for pneumonia.

It is not surprising that inequalities in health and healthcare amongst under-5 children exist in remote rural areas of Nepal as this mirrors similar experience from around the world [29]. The determinants are manifold and have been previously described [30], including key factors such as the accessibility [31] and availability of services [32]. One proposed solution has been the provision of care closer to home that may help address the accessibility issue [33]. However, our study highlights that proximity of services is not enough as even with village-level health posts there remain significant differences in patient health seeking behaviour as well as quality of care provision. Alarmingly, there were high percentages of misdiagnosis of pneumonia and suboptimal treatment subsequently. Ethnic differences were observed with children from the Dalit ethnic group having low ratios for correct diagnosis of pneumonia. Paradoxically, they were more likely to receive correct treatment than the other ethnic groups. Reasons for this are not clear from our data and could be the focus of valuable future research. There was some difference observed in accuracy of diagnosis but not treatment for pneumonia by sex, while the child’s age was not associated with the correct diagnosis or treatment of pneumonia.

### Comparisons to wider literature

As reported elsewhere [34], we found delays in health seeking were associated with district, with patients in less-developed districts in the Far West presenting later than those in the relatively more developed districts in the central region of the country. Likewise, there were greater delays seen for older children than younger children. There were also greater delays in seeking treatment seen for lower caste ethnic groups, which mirrors previously reported lower service utilisation rates for maternal healthcare in Nepal [35, 36]. Part of the explanation for this may be cultural but socio-economic barriers such as poverty cannot be discounted.

The WHO put forward in 2006 the Treat, Train, Retain strategy to address the skills and health workforce shortages in low-income countries [37]. A central pillar of this strategy is task shifting some of the health work to less specialised community health workers. However, the low accuracy of diagnosis and correct treatment of pneumonia even by trained community-level health workers found in our study puts this approach in doubt. Indeed, the figures were not much better compared to reported rates of disease recognition by caregivers [38]. Suboptimal care is likely to lead to poorer health outcomes and undermine efforts to improve child mortality rates in developing countries such as Nepal. Similar issues have been reported elsewhere [39]. This highlights the need for greater efforts, through training and supportive supervision, to improve the clinical performance of frontline community health workers [40].

The other notable finding was that correct diagnoses for pneumonia tended to be made in the presence of a fever. This could reflect the challenge of diagnosing pneumonia based on subtler and more subjective symptoms than fever. This again highlights a training need for frontline staff to better recognise respiratory infections that may not present with a febrile illness.

We also note that disease recognition by health workers showed a two-fold difference between the lowest caste and higher caste patient groups. This cannot be explained by economic or physical access factors. Similarly, there was a gender disparity with more correct diagnoses of pneumonia in males than females. The reasons for both of these observations are not clear and warrant further study.

### What the study adds

Our study demonstrates that there is utility in further and regular analysis of secondary data from primary care settings, including in low- and middle-income countries. The absence of electronic health information systems in rural Nepali health centres inhibits such analysis, with the collection and digitisation of paper-based records being a time-consuming and resource-intensive task. In addition, the analysis of this data is highly dependent on the availability of clinical records that in turn rely on the diligence of health workers recording patient consultations. This data can help inform and guide local service delivery, as well as audit clinical practice as part of service improvement initiatives. However, in keeping with routinely collected clinical data, there will be limitations in terms of accuracy, completeness and timeliness of the data. Some of the limitations are set out below.

## Limitations

There was no consistent tracking of individual patients between visits to a health centre (although there is a field on the form for MRN number, this is often not used - or used inaccurately) so the same children could have visited the health post more than once, yet appear in the data as if they are separate patients. This limitation also meant we could not ascertain the outcomes of consultations beyond prescribing/onward referral. There was also no way to ascertain whether what was recorded by staff was accurately recorded and a true reflection of the clinical picture for the patients attending the clinics.

The determinants of health seeking behaviours, as reflected in the existing literature discussed in the Background section, are multifactorial, and are affected by many things on which the dataset provided no insights, including parents’ level of education; household income; class; and cultural/religious beliefs. Age, sex, ethnicity, and caste are not the only factors influencing these outcomes.

There may also be other confounding factors on the ‘supply side’ that we could not control for and/or that we were not aware of such as staff turnover or potential contaminating effects of local training and supervision that may have taken place. In addition, the study only included data from government health posts that are supported by (or have previously been supported by) PHASE Nepal. Findings may differ in the case of health posts that have not received support from PHASE Nepal, or that have received support from other NGOs.

In our assessment of the diagnostic accuracy of acute respiratory infections, we are making a presumption that the syndromic approach espoused in the IMNCI guidelines is accurate. However, it is recognised that not all febrile illnesses will be due to infections and that there are a wide range of diagnostic possibilities in young children with febrile illnesses.

### What are the implications?

One key insight emerging from our study is the need for good quality monitoring of healthcare activity as part of quality assurance. This is difficult currently based on existing paper records that are not collated or interrogated, and in which it is not possible to reliably track individual children and study follow-up behaviour or health outcomes. Switching from a paper-based record to digital record system may help address these issues, as well as containing fewer recording errors that can occur compared to handwritten records. We have previously demonstrated that it is possible to introduce basic electronic patient record systems even in low-income country settings like Nepal [41].

Our study confirms that there are geographical and socio-demographic inequalities in health-seeking behaviour as well as care provision in rural areas. We identify an important need to raise awareness amongst parents of danger symptoms and signs, especially in certain districts and ethnic groups where awareness levels may be low, so that ill children attend health facilities sooner. We also observed that health workers seem to respond more to some symptoms such as illnesses where there is fever present. They may consequently not appreciate fully serious illnesses where there is no fever. Further training may be needed focused on non-febrile illnesses that may be life-threatening conditions. We also observed a gender bias to health seeking behaviour, with greater delay seen for female children. Therefore, further public education on gender equality remains important.

## Conclusion

The causes of continuing health inequalities in Nepal, and in other low and middle-income countries, are multi-faceted and complex – and relate to a great extent to the social and economic determinants. Nevertheless, demonstrating how differences in geography, sex, ethnicity and caste affect health seeking behaviours and the quality of diagnosis and treatment received following presentation to a health facility is an important step in more fully understanding (and subsequently remedying) inequalities in health service utilisation and quality. The findings of this study identify a range of observed inequalities that can be followed up through detailed qualitative research. The inability to carry out such analysis on an ongoing, routine basis as a result of the absence of electronic record keeping represents an obstacle to identifying and further investigating such inequalities.

## Electronic supplementary material

Below is the link to the electronic supplementary material.

Supplementary Material 1

## Data Availability

The final dataset (with explanatory documentation) is available from the UK Data Service via https://reshare.ukdataservice.ac.uk/854820/.

## Abbreviations

ARI: Acute Respiratory Infection.
GDS: General Danger Signs.
HMIS: Health Management and Information System.
IMCI: Integrated Management of Childhood Illness.
IMNCI: Integrated Management of Neonatal and Childhood Illness.
IQR: Inter-Quartile Range.
MI: Multiple Imputation.
MRN: Master Registration Number.
MUAC: Mid Upper Arm Circumference.
NHRC: Nepal Health Research Council.
NGO: Non-Governmental Organisation.
OR: Odds Ratio.
WHO: World Health Organisation.
