# Supplementary material for: Determinants affecting utilisation of health services and treatment for children under-5 in rural Nepali health centres: a cross-sectional study
Source: BMC Public Health. 2022 Oct 20;22:1948. doi: 10.1186/s12889-022-14318-y (PMC9583555; doi:10.1186/s12889-022-14318-y)
Supplement: Supplementary file 1 — Supplementary Material 1 [file 12889_2022_14318_MOESM1_ESM.docx]

Supplementary Material

Supplementary Figure 1: Copy of IMNCI registers for children aged below 2 months (a) and aged 2 to 59 months (b)

(a)


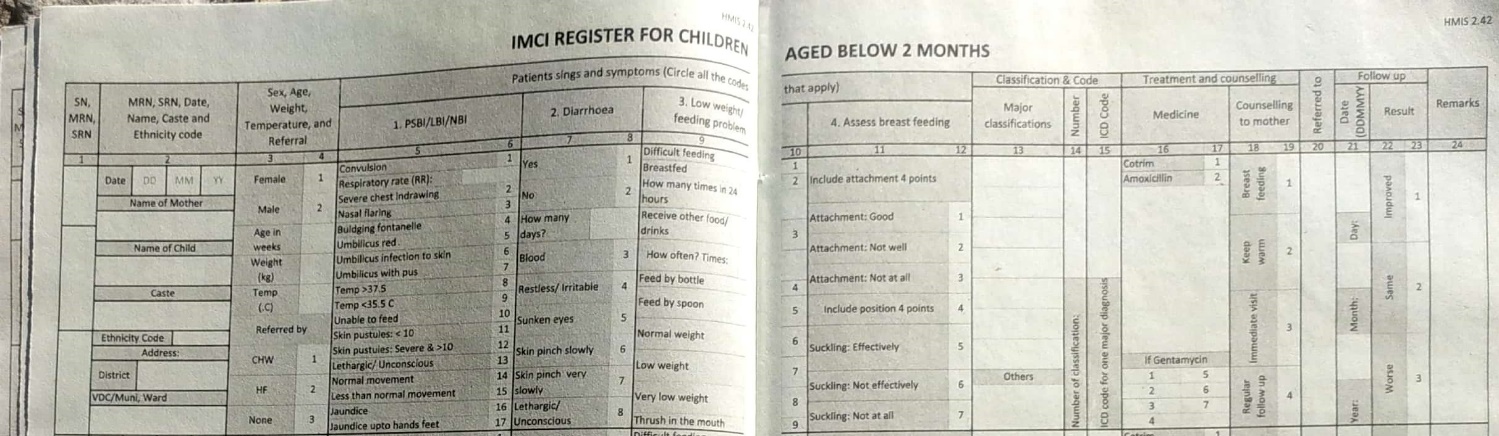


(b)


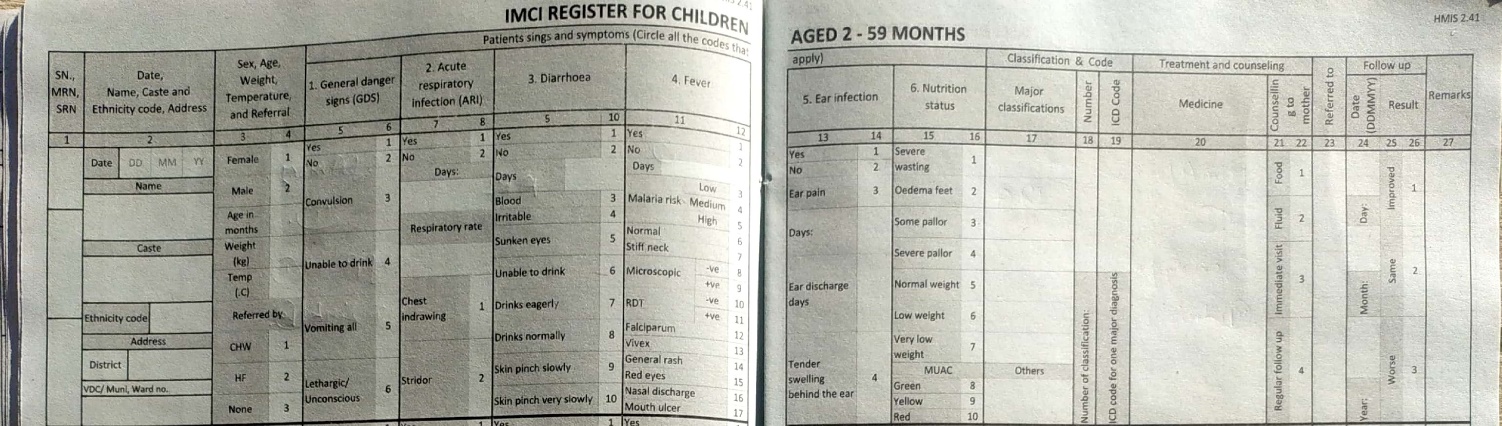


Supplementary Table 1: *Proportion of children who had follow-up, all values frequency with percentage n(%)*

| **Measures** | **Child had a follow-up** | | **p-value** | **Measures** | **Child had a follow-up** | | **p-value** |
| --- | --- | --- | --- | --- | --- | --- | --- |
|  | **No** | **Yes** |  |  | **No** | **Yes** |  |
| **District** |  |  | <0.001 | **GDS** |  |  | 0.657 |
| Sindhupalchowk | 3692 (94.3) | 223 (5.7) |  | No | 523 (96.7) | 18 (3.3) |  |
| Gorkha | 6960 (95.8) | 304 (4.2) |  | Yes | 25260 (96.3) | 968 (3.7) |  |
| Humla | 3290 (97.9) | 72 (2.1) |  | **ARI** |  |  | <0.001 |
| Mugu | 3980 (94.4) | 236 (5.6) |  | No | 9223 (94.6) | 525 (5.4) |  |
| Bajura | 11659 (97.4) | 315 (2.6) |  | Yes | 17344 (97.1) | 522 % (2.9) |  |
| **Ethnicity** |  |  | <0.001* | **Diarrhoea** |  |  | 0.9590 |
| Dalit | 6176 (96.5) | 223 (3.5) |  | No | 10127 (96.2) | 395 (3.8) |  |
| Janajati | 10207 (95.5) | 485 (4.5) |  | Yes | 16712 (96.2) | 654 (3.8) |  |
| Madhesi | 157 (98.7) | 2 (1.3) |  | **Ear Infection** |  |  | 0.091 |
| Muslim | 69 (97.2) | 2 (2.8) |  | No | 1524 (97) | 47 (3) |  |
| Brahmin/ Chhetri | 9765 (96.9) | 308 (3.1) |  | Yes | 22671 (96.2) | 903 (3.8) |  |
| Others | 2859 (95.9) | 123 (4.1) |  | **Fever** |  |  | <0.001 |
| **Sex** |  |  | 0.566 | Yes | 11089 (95.1) | 568 (4.9) |  |
| Female | 13092 (96.3) | 500 (3.7) |  | No | 15056 (97.2) | 430 (2.8) |  |
| Male | 16437 (96.2) | 650 (3.8) |  | **Total** | 29581 (96.3) | 1150 (3.7) |  |
| **Referred by** |  |  | <0.001* |  | | | |
| CHW | 71 (88.8) | 9 (11.3) |  |  |  |  |  |
| HF | 854 (99) | 9 (1) |  |  |  |  |  |
| None | 9904 (95.9) | 421 (4.1) |  |  |  |  |  |
| FCHV | 15 (100) | 0 (0) |  |  |  |  |  |
| PHC/ORC | 39 (100) | 0 (0) |  |  |  |  |  |

*Footnote: ARI= Acute Respiratory Infection; CHW= Community Health Worker; FCHV= Female Community Health Worker; GDS= General Danger Sign; HF= Health Facility; ORC= Outreach Clinic; PHC= Primary Health Centre; All p-values Chi-square test unless marked with a * (*=Fishers Exact Test).*

Supplementary Table 2: *Univariable and multivariable linear regression of number of ARI days using complete case analysis*

|  | ARI (n=2498) | | | | | |
| --- | --- | --- | --- | --- | --- | --- |
|  | **Coef** | **95% CI** | **P-Value** | **Adj Coef** | **95% CI** | **P-Value** |
| **Health facility district** |  |  |  |  |  |  |
| Gorkha | REF |  |  | REF |  |  |
| Sindhupalchowk | 0.39 | -0.37, 1.14 | 0.316 | 0.71 | 0.10, 1.31 | 0.023 |
| Humla | 2.97 | -0.33, 6.27 | 0.078 | 1.34 | 0.62, 2.05 | <0.001 |
| Mugu | 1.16 | 0.11, 2.21 | 0.031 | 1.70 | 0.68, 2.73 | 0.001 |
| Bajura | 0.33 | -0.33, 1.00 | 0.327 | 0.71 | 0.10, 1.33 | 0.023 |
| **Visit year Nepali year** |  |  |  |  |  |  |
| 2070 to 2073 | REF |  |  | REF |  |  |
| 2074 to 2076 | -0.25 | -0.56, 0.07 | 0.121 | -0.26 | -0.56, 0.03 | 0.079 |
| **Ethnicity** |  |  |  |  |  |  |
| Dalit | REF |  |  | REF |  |  |
| Janajati | -0.37 | -0.80, 0.06 | 0.091 | 0.21 | -0.30, 0.71 | 0.424 |
| Madhesi | 3.41 | 1.51, 5.31 | <0.001 | 1.02 | -1.24, 3.28 | 0.375 |
| Muslim | -1.11 | -2.33, 0.10 | 0.073 | -0.57 | -0.83, -0.30 | <0.001 |
| Brahmin/Chhetri | 0.32 | 0.00, 0.64 | 0.048 | 0.31 | -0.12, 0.74 | 0.157 |
| Others | -0.03 | -0.43, 0.37 | 0.887 | 0.13 | -0.72, 0.98 | 0.763 |
| **Sex** |  |  |  |  |  |  |
| Female | REF |  |  | REF |  |  |
| Male | 0.07 | -0.19, 0.32 | 0.603 | 0.90 | 0.31, 1.49 | 0.003 |
| **Age (months)** | -0.01 | -0.02, 0.01 | 0.314 | -0.01 | -0.02, 0.01 | 0.294 |
| **Temperature (°C)** | -0.16 | -0.56, 0.24 | 0.437 |  |  |  |
| **Referred** |  |  |  |  |  |  |
| No | REF |  |  | REF |  |  |
| Yes | -0.49 | -1.14, 0.15 | 0.133 | -0.48 | -1.02, 0.07 | 0.085 |
| **GDS** |  |  |  |  |  |  |
| No | REF |  |  |  |  |  |
| Yes | 0.79 | -0.60, 2.18 | 0.266 |  |  |  |
| **Diarrhoea** |  |  |  |  |  |  |
| No | REF |  |  |  |  |  |
| Yes | 0.26 | -0.37, 0.89 | 0.422 |  |  |  |
| **Ear Infection** |  |  |  |  |  |  |
| No | REF |  |  |  |  |  |
| Yes | -0.31 | -0.88, 0.26 | 0.290 |  |  |  |
| **Fever** |  |  |  |  |  |  |
| No | REF |  |  |  |  |  |
| Yes | -0.12 | -0.56, 0.32 | 0.604 |  |  |  |
| **Interaction: Gender*District** |  |  |  |  |  |  |
| Male Sindhupalchowk |  |  |  | -1.19 | -1.85, -0.52 | <0.001 |
| Male Humla |  |  |  | 0.00 | -0.93, 0.93 | 0.993 |
| Male Mugu |  |  |  | -0.72 | -1.39, -0.04 | 0.038 |
| Male Bajura |  |  |  | -0.71 | -1.40, -0.02 | 0.042 |
| **Interaction: Gender*Ethnicity** |  |  |  |  |  |  |
| Male Janajati |  |  |  | -0.52 | -0.96, -0.08 | 0.020 |
| Male Madhesi |  |  |  | 0.00 | -0.29, 0.28 | 0.987 |
| Male Muslim |  |  |  | 0.00 | -0.29, 0.28 | 0.987 |
| Male Brahmin/Chhetri |  |  |  | 0.03 | -0.61, 0.67 | 0.928 |
| Male Others |  |  |  | 0.05 | -0.21, 0.31 | 0.695 |

*Footnote: Also adjusted for visit year; ARI= Acute Respiratory Infection; CI= Confidence Interval;* °C*= degrees Celsius; GDS= General Danger Sign; REF= Reference category.*

Supplementary Table 3: *Univariable and multivariable linear regression of number of diarrhoea days using complete case analysis*

|  | Diarrhoea (n=2115*) | | | | | |
| --- | --- | --- | --- | --- | --- | --- |
|  | **Coef** | **95% CI** | **P-Value** | **Adj Coef** | **95% CI** | **P-Value** |
| **Health facility district** |  |  |  |  |  |  |
| Gorkha | REF |  |  | REF |  |  |
| Sindhupalchowk | 0.90 | -0.31, 2.11 | 0.145 | 1.64 | -0.60, 3.88 | 0.152 |
| Humla | 2.06 | 1.67, 2.46 | <0.001 | 2.14 | 1.20, 3.08 | <0.001 |
| Mugu | 1.46 | 0.86, 2.07 | <0.001 | 1.55 | 0.59, 2.51 | 0.002 |
| Bajura | 1.13 | 0.42, 1.84 | 0.002 | 1.07 | -0.10, 2.24 | 0.073 |
| **Visit year Nepali year** |  |  |  |  |  |  |
| 2070 to 2073 | REF |  |  | REF |  |  |
| 2073 to 2076 | -0.10 | -0.70, 0.50 | 0.734 | -0.14 | -0.69, 0.41 | 0.626 |
| **Ethnicity** |  |  |  |  |  |  |
| Dalit | REF |  |  | REF |  |  |
| Janajati | -1.10 | -1.93, -0.26 | 0.010 | 0.07 | -0.66, 0.80 | 0.852 |
| Madhesi | -1.09 | -1.96, -0.22 | 0.014 | -0.82 | -1.86, 0.21 | 0.119 |
| Muslim | Insufficient sample size | | | Insufficient sample size | | |
| Brahmin/Chhetri | -0.31 | -0.69, 0.06 | 0.103 | -0.31 | -0.62, -0.00 | 0.049 |
| Others | -0.03 | -0.58, 0.51 | 0.913 | -0.13 | -0.64, 0.39 | 0.629 |
| **Sex** |  |  |  |  |  |  |
| Female | REF |  |  | REF |  |  |
| Male | -0.09 | -0.45, 0.27 | 0.620 | -0.49 | -0.94, -0.05 | 0.030 |
| **Age (months)** | -0.01 | -0.04, 0.01 | 0.366 | -0.01 | -0.03, 0.02 | 0.600 |
| **Temperature (°C)** | -0.10 | -0.47, 0.27 | 0.585 |  |  |  |
| **Referred** |  |  |  |  |  |  |
| No | REF |  |  | REF |  |  |
| Yes | -0.57 | -1.07, -0.06 | 0.027 | -0.54 | -1.02, -0.06 | 0.028 |
| **GDS** |  |  |  |  |  |  |
| No | REF |  |  |  |  |  |
| Yes | 1.78 | -4.08, 7.64 | 0.552 |  |  |  |
| **ARI** |  |  |  |  |  |  |
| No | REF |  |  |  |  |  |
| Yes | 0.03 | -0.58, 0.65 | 0.913 |  |  |  |
| **Ear Infection** |  |  |  |  |  |  |
| No | REF |  |  |  |  |  |
| Yes | -0.10 | -0.92, 0.72 | 0.806 |  |  |  |
| **Fever** |  |  |  |  |  |  |
| No | REF |  |  | REF |  |  |
| Yes | 0.36 | -0.13, 0.85 | 0.154 | 0.25 | -0.21, 0.71 | 0.283 |
| **Interaction: Gender*District** |  |  |  |  |  |  |
| Male Sindhupalchowk |  |  |  | -1.42 | -3.61, 0.77 | 0.204 |
| Male Humla |  |  |  | 0.16 | -0.79, 1.10 | 0.747 |
| Male Mugu |  |  |  | 0.24 | -0.35, 0.83 | 0.431 |
| Male Bajura |  |  |  | 0.66 | 0.03, 1.29 | 0.039 |

*Footnote: Also adjusted for visit year; ARI= Acute Respiratory Infection; CI= Confidence Interval;* °C*= degrees Celsius; GDS= General Danger Sign; REF= Reference category.*

Supplementary Table 4: *Univariable and multivariable linear regression of number of fever days using complete case analysis*

|  | Fever (n=2462) | | | | | |
| --- | --- | --- | --- | --- | --- | --- |
|  | **Coef** | **95% CI** | **P-Value** | **Adj Coef** | **95% CI** | **P-Value** |
| **District** |  |  |  |  |  |  |
| Gorkha | REF |  |  | REF |  |  |
| Sindhupalchowk | 0.03 | -0.17, 0.22 | 0.784 | -0.04 | -0.26, 0.18 | 0.711 |
| Humla | 0.81 | 0.56, 1.06 | <0.001 | 0.73 | 0.30, 1.15 | 0.001 |
| Mugu | 0.90 | 0.62, 1.17 | <0.001 | 0.88 | 0.40, 1.37 | <0.001 |
| Bajura | 0.76 | 0.56, 0.97 | <0.001 | 0.65 | 0.22, 1.08 | 0.003 |
| **Visit year Nepali year** |  |  |  |  |  |  |
| 2070 to 2073 | REF |  |  | REF |  |  |
| 2073 to 2076 | 0.14 | -0.17, 0.46 | 0.366 | 0.17 | -0.13, 0.47 | 0.269 |
| **Ethnicity** |  |  |  |  |  |  |
| Dalit | REF |  |  | REF |  |  |
| Janajati | -0.79 | -1.02, -0.56 | <0.001 | -0.19 | -0.53, 0.15 | 0.281 |
| Madhesi | -0.37 | -0.74, 0.00 | 0.050 | -0.50 | -0.85, -0.14 | 0.006 |
| Muslim | -0.47 | -1.23, 0.29 | 0.225 | -1.32 | -1.69, -0.95 | <0.001 |
| Brahmin/Chhetri | -0.02 | -0.25, 0.20 | 0.835 | -0.01 | -0.33, 0.31 | 0.951 |
| Others | -0.28 | -0.53, -0.03 | 0.031 | -0.28 | -0.66, 0.09 | 0.134 |
| **Sex** |  |  |  |  |  |  |
| Female | REF |  |  | REF |  |  |
| Male | -0.08 | -0.19, 0.03 | 0.179 | -0.06 | -0.32, 0.20 | 0.647 |
| **Age (months)** | 0.01 | 0.00, 0.01 | 0.009 | 0.01 | 0.00, 0.01 | 0.001 |
| **Temperature (°C)** | 0.01 | -0.08, 0.10 | 0.858 |  |  |  |
| **Referred** |  |  |  |  |  |  |
| No | REF |  |  |  |  |  |
| Yes | -0.16 | -0.44, 0.12 | 0.269 |  |  |  |
| **GDS** |  |  |  |  |  |  |
| No | REF |  |  |  |  |  |
| Yes | 0.02 | -0.48, 0.51 | 0.943 |  |  |  |
| **ARI** |  |  |  |  |  |  |
| No | REF |  |  |  |  |  |
| Yes | 0.12 | -0.14, 0.38 | 0.364 |  |  |  |
| **Diarrhoea** |  |  |  |  |  |  |
| No | REF |  |  | REF |  |  |
| Yes | 0.15 | -0.03, 0.34 | 0.111 | 0.05 | -0.15, 0.25 | 0.603 |
| **Ear Infection** |  |  |  |  |  |  |
| No | REF |  |  |  |  |  |
| Yes | -0.05 | -0.35, 0.25 | 0.738 |  |  |  |
| **Interaction: Gender*Ethnicity** |  |  |  |  |  |  |
| Male Janajati |  |  |  | 0.00 | -0.29, 0.30 | 0.979 |
| Male Madhesi |  |  |  | 0.03 | -0.46, 0.53 | 0.890 |
| Male Muslim |  |  |  | 1.05 | 0.71, 1.39 | <0.001 |
| Male Brahmin/Chhetri |  |  |  | -0.08 | -0.41, 0.25 | 0.635 |
| Male Others |  |  |  | -0.21 | -0.54, 0.12 | 0.207 |

*Footnote: Also adjusted for visit year; ARI= Acute Respiratory Infection; CI= Confidence Interval;* °C*= degrees Celsius; GDS= General Danger Sign; REF= Reference category.*

Supplementary Table 5: *Unadjusted and adjusted logistic regression results of correct diagnosis of pneumonia vs not (reference) and correct treatment of pneumonia vs not (reference) using complete case analysis*

|  | **Correct diagnosis of Pneumonia (n=753*)** | | | | | | **Correct treatment of Pneumonia (n=525*)** | | | | | |
| --- | --- | --- | --- | --- | --- | --- | --- | --- | --- | --- | --- | --- |
|  | **OR** | **95% CI** | **P-Value** | **AOR** | **95% CI** | **P-Value** | **OR** | **95% CI** | **P-Value** | **AOR** | **95% CI** | **P-Value** |
| **District** |  |  |  |  |  |  |  |  |  |  |  |  |
| Gorkha | REF |  |  | REF |  |  | REF |  |  | REF |  |  |
| Sindhupalchowk | 2.56 | 0.75, 8.74 | 0.135 | 2.54 | 0.51, 12.55 | 0.253 | 14.42 | 1.34, 155.41 | 0.028 | 16.30 | 1.34, 197.87 | 0.028 |
| Humla | 1.30 | 0.13, 13.24 | 0.825 | 1.54 | 0.14, 17.44 | 0.729 | 6.02 | 0.04, 983.99 | 0.490 | 3.74 | 0.01, 930.34 | 0.640 |
| Mugu | 6.63 | 1.45, 30.40 | 0.015 | 6.59 | 1.05, 41.23 | 0.044 | 14.40 | 0.88, 236.68 | 0.062 | 9.70 | 0.43, 218.13 | 0.153 |
| Bajura | 1.52 | 0.45, 5.14 | 0.501 | 1.32 | 0.21, 8.53 | 0.767 | 3.99 | 0.85, 18.69 | 0.079 | 2.67 | 0.43, 16.43 | 0.289 |
| **Visit year Nepali year** |  |  |  |  |  |  |  |  |  |  |  |  |
| 2070 to 2073 | REF |  |  | REF |  |  | REF |  |  | REF |  |  |
| 2073 to 2076 | 0.73 | 0.34, 1.56 | 0.419 | 0.73 | 0.23, 2.34 | 0.598 | 1.07 | 0.42, 2.71 | 0.891 | 1.02 | 0.37, 2.80 | 0.964 |
| **Ethnicity** |  |  |  |  |  |  |  |  |  |  |  |  |
| Dalit | REF |  |  | REF |  |  | REF |  |  | REF |  |  |
| Janajati | 0.97 | 0.47, 2.02 | 0.937 | 1.16 | 0.53, 2.55 | 0.707 | 0.58 | 0.25, 1.37 | 0.213 | 0.63 | 0.29, 1.37 | 0.246 |
| Madhesi | 0.69 | 0.04, 13.07 | 0.807 | 1.46 | 0.19, 11.35 | 0.719 | 1.06 | 0.76, 1.48 | 0.750 | 1.18 | 0.61, 2.26 | 0.628 |
| Muslim | Insufficient sample size | | | | | | Insufficient sample size | | | | | |
| Brahmin/Chhetri | 1.02 | 0.69, 1.51 | 0.910 | 1.14 | 0.82, 1.58 | 0.430 | 1.17 | 0.69, 1.98 | 0.571 | 1.26 | 0.72, 2.24 | 0.419 |
| Others | 0.84 | 0.37, 1.90 | 0.679 | 0.85 | 0.35, 2.07 | 0.717 | 0.45 | 0.25, 0.82 | 0.009 | 0.42 | 0.24, 0.73 | 0.002 |
| **Sex** |  |  |  |  |  |  |  |  |  |  |  |  |
| Female | REF |  |  | REF |  |  | REF |  |  | REF |  |  |
| Male | 1.40 | 0.78, 2.51 | 0.265 | 2.49 | 1.46, 4.25 | 0.001 | 1.33 | 0.81, 2.21 | 0.263 | 1.48 | 0.92, 2.39 | 0.106 |
| **Age (months)** | 1.00 | 0.98, 1.01 | 0.415 | 0.99 | 0.98, 1.00 | 0.172 | 1.00 | 0.98, 1.02 | 0.971 | 1.00 | 0.98, 1.02 | 0.993 |
| **Temperature (°C)** | 1.41 | 0.99, 2.01 | 0.054 | 1.50 | 1.02, 2.21 | 0.038 | 1.06 | 0.83, 1.34 | 0.651 |  |  |  |
| **Referred** |  |  |  |  |  |  |  |  |  |  |  |  |
| No | REF |  |  |  |  |  | REF |  |  |  |  |  |
| Yes | 0.55 | 0.39, 0.79 | 0.001 | 0.59 | 0.36, 0.97 | 0.038 | 1.71 | 1.14, 2.56 | 0.010 | 1.95 | 1.18, 3.21 | 0.009 |
| **GDS** |  |  |  |  |  |  |  |  |  |  |  |  |
| No | REF |  |  | REF |  |  | REF |  |  |  |  |  |
| Yes | 0.02 | 0.00, 0.14 | <0.001 | 0.01 | 0.00, 0.07 | <0.001 | 0.48 | 0.13, 1.79 | 0.275 |  |  |  |
| **ARI** |  |  |  |  |  |  |  |  |  |  |  |  |
| No | REF |  |  |  |  |  | REF |  |  |  |  |  |
| Yes | 0.38 | 0.11, 1.33 | 0.129 | 0.27 | 0.07, 1.01 | 0.051 | 2.62 | 0.09, 77.62 | 0.578 |  |  |  |
| **Diarrhoea** |  |  |  |  |  |  |  |  |  |  |  |  |
| No | REF |  |  |  |  |  | REF |  |  | REF |  |  |
| Yes | 1.26 | 0.84, 1.89 | 0.268 |  |  |  | 1.57 | 0.96, 2.59 | 0.074 | 1.65 | 1.17, 2.34 | 0.004 |
| **Ear Infection** |  |  |  |  |  |  |  |  |  |  |  |  |
| No | REF |  |  | REF |  |  | REF |  |  | REF |  |  |
| Yes | 0.77 | 0.40, 1.50 | 0.447 |  |  |  | 0.37 | 0.13, 1.10 | 0.075 | 0.34 | 0.10, 1.13 | 0.079 |
| **Fever** |  |  |  |  |  |  |  |  |  |  |  |  |
| No | REF |  |  | REF |  |  | REF |  |  | REF |  |  |
| Yes | 1.98 | 1.09, 3.62 | 0.026 | 3.26 | 1.70, 6.25 | <0.001 | 1.96 | 0.89, 4.31 | 0.093 | 2.11 | 0.88, 5.06 | 0.096 |
| **Interaction: Gender*GDS** |  |  |  |  |  |  |  |  |  |  |  |  |
| Male*GDS |  |  |  | 5.71 | 1.54, 21.17 | 0.009 |  |  |  |  |  |  |
| **Interaction: Gender*Fever** |  |  |  |  |  |  |  |  |  |  |  |  |
| Male*Fever |  |  |  | 0.38 | 0.19, 0.75 | 0.005 |  |  |  |  |  |  |

*Footnote: Also adjusted for visit year; Muslim removed due to sample size; AOR= Adjusted Odds Ratio; ARI= Acute Respiratory Infection; CI= Confidence Interval;* °C*= degrees Celsius; GDS= General Danger Sign; OR= Odds Ratio; REF= Reference category.*
